# Hypoxic Living and Exercise Training Alter Adipose Tissue Leptin/Leptin Receptor in Rats

**DOI:** 10.3389/fphys.2016.00554

**Published:** 2016-11-23

**Authors:** Yingli Lu, Lianshi Feng, Minhao Xie, Li Zhang, Jianfang Xu, Zihong He, Tongjian You

**Affiliations:** ^1^China Institute of Sport ScienceBeijing, China; ^2^China Institute of Sports MedicineBeijing, China; ^3^Department of Exercise and Health Sciences, College of Nursing and Health Sciences, University of Massachusetts BostonBoston, MA, USA

**Keywords:** hypoxia, exercise, adipose tissue, leptin, leptin receptor

## Abstract

**Background:** Hypobaric hypoxia results in weight loss in obese individuals, and exercise training is advocated for the treatment of obesity and its related metabolic dysfunctions. The purpose of this study was to investigate the effects of hypoxic living and exercise training on obesity and adipose tissue leptin/leptin receptor in dietary-induced obese rats.

**Methods:** One hundred and thirty high-fat diet fed Sprague-Dawley rats were assigned into one of the following groups (*n* = 10 each): control, sedentary hypoxic living for 1–4 weeks (SH1, SH2, SH3, and SH4), living, and exercise training in normoxic conditions for 1–4 weeks (TN1, TN2, TN3, and TN4), and living and exercise training in hypoxic conditions for 1–4 weeks (TN1, TN2, TN3, and TN4). Epididymal adipose tissue expression levels of leptin and leptin receptor were determined

**Results:** Compared to hypoxic living and living and exercise training in normoxic conditions, living and exercise training in hypoxic conditions for 3–4 weeks resulted in lower Lee index (*P* < 0.05–0.01), and higher expression of leptin and leptin receptor (*P* < 0.05–0.01) in adipose tissue.

**Conclusion:** In a rodent model of altitude training, living, and exercise training in hypoxic conditions resulted in greater alterations in obesity and adipose tissue leptin/leptin receptor than hypoxic living alone and living and exercise training in normoxic conditions.

## Introduction

Obesity is a global public health issue, and is associated with a higher risk of type 2 diabetes and cardiovascular disease. Hypocaloric diet and exercise training have been advocated for obesity treatments. Human studies have shown that hypocaloric diet alone, or diet plus exercise, is effective in lowering adiposity and improve obesity-related metabolic dysfunctions (You et al., 2004; Campbell et al., 2013). Several studies indicate that exercise training alone also lowers obesity and improves metabolic dysfunctions through its effects on lipid metabolism and inflammation (Ross et al., 2000; You et al., 2004; Lazzer et al., 2011).

Moderate altitude living results in weight loss and changes in metabolic functions in obese individuals (Lippl et al., 2010). Current proposed mechanisms include a reduction of energy intake, a reduction of intestinal energy uptake, and an increased energy expenditure (Kayser and Verges, 2013). It has been postulated that activation of hypoxia inducible factor (HIF) could potentially leads to several metabolic benefits in obese individuals living at altitude (Palmer and Clegg, 2014). Hypoxia also induces changes in adipocytes in the expression and release of adipokines, such as leptin (Tschop et al., 1998; Shukla et al., 2005), which plays an important role in the regulation of energy metabolism (Ahima and Flier, 2000). However, reports in the literature present contradictory findings, presumably attributable to different experimental designs (Sierra-Johnson et al., 2008).

Normobaric hypoxia training results in more weight loss than normoxia training in obese adults (Kong et al., 2014). Interestingly, daily moderate exercise training during 10-day continuous hypoxic exposure improved lipid profile, but did not alter hormonal appetite regulation, including fasting levels of leptin, in healthy young men (Debevec et al., 2014). However, fasting leptin levels were reduced following daily moderate exercise training during 16-day continuous hypoxic exposure with no changes observed following bed rest in normoxia and bad rest in hypoxia (Debevec et al., 2016).

It would be of importance to know if living and exercise training in hypoxic conditions can result in additional benefits than hypoxic living alone or living and exercise training in normoxic conditions, as this will help identify novel weight loss strategies. Therefore, the purpose of this study was to investigate the effects of hypoxic living and exercise training on obesity and adipose tissue leptin/leptin receptor in dietary-induced obese rats. Our hypothesis was that, compared to hypoxic living, and exercise training in normoxic conditions, living, and exercise training in hypoxic conditions would result in greater alterations in body composition and adipose tissue leptin/leptin receptor in obese rats.

## Materials and methods

### Animals and procedures

This study protocol was approved by the animal use committee at China Institute of Sport Science. One hundred and thirty male Sprague—Dawley rats were purchased from Vital River Laboratories (Beijing, China) and were fed a high-fat diet for 3 months and then randomized into one of the following 13 groups as previously described (He et al., 2012): C (control): sedentary living in normoxic conditions for 2 days; SH1, SH2, SH3, and SH4: sedentary living in normobaric hypoxic conditions for 1–4 weeks, respectively; TN1, TN2, TN3, and TN4: living and training in normoxic conditions for 1–4 weeks, respectively; TH1, TH2, TH3, and TH4: living and training in normobaric hypoxia conditions for 1–4 weeks, respectively.

All groups had free access to high-fat chow and water. In SH1–SH4 and TH1–TH4 groups, rats were exposed for 24 h per day to hypoxic conditions with a constant O_2_ concentration of 13.6% O_2_ in breathing air (corresponding to an altitude of 3500 m), which was made by air compressors (GA15FF-13, Atlas Copco Stockholm, Sweden) and a nitrogen generator (CA-200AT, CNRO, Tianjin, China). Rats in groups TN1–TN4 were exercised continuously for 1 h per day, 6 days per week on a rat treadmill at a speed of 26 m/min and an incline of 0°C. Rats in groups TH1–TH4 were kept to exercise in a similar protocol except for walking at a speed of 21 m/min and an incline of 0°C. These speeds correspond to similar exercise intensities according to the measured mean blood lactate concentrations (~3 mmol/L) (He et al., 2012).

After an overnight fast, all rats were measured for the parameters of body length and weight at the same time of the week (38 h after the last bout of exercise for the exercise training groups). Lee index was calculated using the following formula: Lee index = body weight(g)^1/3^/ body length (cm) × 1000. Rats were anesthetized by intraperitoneal injection of 10% chloral hydrate solution. Blood samples were drawn from abdominal aorta and then centrifuged at 3000 r/min for 15 min. Serum samples were collected and stored at −80°C for measuring serum leptin levels. The left perirenal and epididymal fat pads were quickly separated and weighed, then frozen in liquid nitrogen and stored at −80°C for measuring levels of leptin and leptin receptor.

About 0.2 g epididymal adipose tissue per sample was grinded in a precooled mortar. The tissue was transferred into centrifugal tube added 0.5 ml tissue protein lysis buffer, which contained phenylmethylsulfonyl fluoride. The tube was vortexed and then centrifuged at a speed of 12,000 r/min and a temperature of 4°C for 15 min. The intranatant was collected for measuring leptin and leptin receptor. Adipose tissue levels of leptin and leptin receptor, and serum levels of leptin were measured by using enzyme-linked-immunosorbent assay (ELISA) kits (R&D Systems, Minneapolis, USA). The intra- and inter-assay coefficient of variation values for the leptin assay were 5.1 and 64%, respectively; and the intra- and inter-assay coefficient of variation values for the leptin receptor assay were 7.2 and 8.0%, respectively.

### Statistical analyses

Data analyses were performed by using one-way analysis of variance (ANOVA) to evaluate mean differences among the groups. Tukey *post-hoc* tests were used for individual group comparisons. All results are expressed as mean ± standard deviation. Statistical significance was set at *p* < 0.05 for all analyses.

## Results

Group comparisons in (perirenal and epididymal) fat weights and Lee index are shown in Table 1. There were significant ANOVA main effects on the group comparisons for the perirenal fat weight and epididymal fat weight (both *p* < 0.001). Compared to that of the control group, perirenal fat weight or epididymal fat weight was not different in each of the SH1-SH4, TN1-TN4, and TH1-TH4 groups; perirenal fat weight or epididymal fat weight in each TH group was not significantly different compared to each corresponding TN group. Perirenal fat weight values in the TN2-TN4 and TH3-TH4 groups were significantly lower compared to corresponding SH groups (*p* < 0.01–0.05). Epididymal fat weight values in the TN4 groups were significantly lower compared to corresponding SH groups (all *p* < 0.05). There was a significant ANOVA main effect on the group comparisons for the Lee index (*p* < 0.001). Lee index was significantly lower in each of the SH2-SH4, TN3-TN4, and TH2-TH4 groups compared to that of the control group (*p* < 0.05–0.001). Lee index in each of the TH3-TH4 groups was significantly lower compared to each of the corresponding SH and TN groups (*p* < 0.05–0.01).

**Table 1:** **Effects of hypoxic living and exercise training on fat weight and Lee index in rats**.

	**Perirenal fat weight (g)**	**Epididymal fat weight (g)**	**Lee index**
C	1.40 ± 0.42	4.64 ± 1.68	325.88 ± 10.79
SH1	1.64 ± 0.66	4.26 ± 1.33	322.08 ± 8.41
SH2	2.07 ± 0.50	4.63 ± 1.29	315.29 ± 6.78^*^
SH3	2.17 ± 0.53	5.42 ± 1.24	314.03 ± 2.98^**^
SH4	2.03 ± 0.51	5.24 ± 1.10	312.24 ± 6.16^**^
TN1	1.31 ± 0.53	4.17 ± 1.01	316.70 ± 5.39
TN2	1.35 ± 0.28#	4.25 ± 0.83	316.26 ± 3.98
TN3	1.36 ± 0.32##	4.16 ± 2.05	309.94 ± 6.94^**^
TN4	1.07 ± 0.41##	3.41 ± 1.18#	308.27 ± 4.88^**^
TH1	1.24 ± 0.30	3.73 ± 0.97	320.91 ± 9.30
TH2	1.52 ± 0.24	3.29 ± 0.63	309.28 ± 5.18^**^
TH3	1.39 ± 0.39#	3.68 ± 1.41#	303.65 ± 7.98^**^#&
TH4	1.37 ± 0.60#	3.79 ± 1.57#	302.73 ± 4.82^**^##&

* *p < 0.05*,

** *p < 0.01 vs. C group*,

# *p < 0.05*,

## *p < 0.01 vs. SH group*,

& *p < 0.05 vs. TN group*.

Group comparisons in adipose tissue leptin, adipose tissue lepin receptor, and serum leptin levels are shown in Table 2. There was a significant main effect on the group comparisons for adipose tissue leptin (*p* < 0.001). Compared to those of the control group, adipose tissue leptin levels were lower in the TN4 groups (*p* < 0.01), and higher in the TH3-TH4 groups (both *p* < 0.01). Leptin levels in the TH2-TH4 groups were significantly higher compared to those in the corresponding SH and TN groups (*p* < 0.01–0.001). There was a significant main effect on the group comparisons for adipose tissue leptin receptor (*p* < 0.001). Compared to those of the control group, adipose tissue leptin receptor levels were higher in, and TH2-TH4 groups (*p* < 0.05–0.001); Leptin receptor levels were higher in the TH3-TH4 groups compared to corresponding SH and TN groups (all *p* < 0.05). There was not a significant main effect on the group comparisons for serum leptin levels (*p* = 0.338). Therefore, there were no significant differences among all groups.

**Table 2:** **Effects of hypoxic living and exercise training on epididymal adipose tissue leptin/leptin receptor levels in rats**.

	**Adipose tissue leptin (ug/L)**	**Adipose tissue leptin receptor (ng/ml)**	**Serum leptin (pg/ml)**
C	1.08 ± 0.27	5.48 ± 1.12	632.8 ± 83.9
SH1	1.05 ± 0.47	7.91 ± 1.59	631.0 ± 25.3
SH2	0.80 ± 0.28	6.47 ± 1.98	667.1 ± 61.4
SH3	0.81 ± 0.27	7.98 ± 2.13	682.4 ± 82.0
SH4	0.83 ± 0.18	6.78 ± 2.03	691.2 ± 107.1
TN1	0.79 ± 0.29	6.70 ± 2.33	636.2 ± 93.3
TN2	0.71 ± 0.29	7.13 ± 3.22	664.2 ± 78.1
TN3	0.75 ± 0.30	6.44 ± 1.61	672.0 ± 127.7
TN4	0.38 ± 0.28^**^	7.69 ± 1.45	691.3 ± 143.4
TH1	1.07 ± 0.24	8.35 ± 3.11	664.2 ± 95.9
TH2	1.54 ± 0.31##&&	8.74 ± 1.81^*^	704.8 ± 111.3
TH3	1.81 ± 0.49^**^##&&	9.41 ± 1.32^*^#&	719.5 ± 53.9
TH4	1.78 ± 0.45^**^##&&	10.48 ± 2.23^**^#&	729.4 ± 45.1

* *p < 0.05*,

** *p < 0.01 vs. C group*,

# *p < 0.05*,

## *p < 0.01 vs. SH group*,

& *p < 0.05*,

&& *p < 0.01 vs. TN group*.

## Discussion

Our study is the first one to report that hypoxic living plus exercise training, compared to either hypoxic living alone or living and exercise training in normoxic conditions, resulted in lower adiposity, and higher levels of an adipose tissue derived energy-regulating hormone and its receptor.

Previous human studies indicated that altitude living lowered body weight in obese subjects (Lippl et al., 2010). Interestingly, leptin levels increased in high altitude despite reduced body weight. Hypobaric hypoxia seemed to play a major role in causing weight changes. Although the authors did observe changes in metabolic rate and food intake, the exact physiological mechanisms remained unclear. It has been reported that the greatest effects were observed within the first 3 months of the hypoxic exposure, with only slight changes thereafter. This may suggest a potential stabilization of the effects after 3 months (Gatterer et al., 2015). The majority of studies have showed a decrease in plasma leptin levels with (Bailey et al., 2004) and without (Woolcott et al., 2002) significant weight reduction at high altitude, whereas other studies reported unchanged plasma leptin levels with (Debevec et al., 2014) and without (Barnholt et al., 2006) a change in body weight or adiposity. In animal studies, hypoxia exposure lowered body weight and also increased leptin expression in adipose tissue (Simler et al., 2006). Hypoxia also led to an increase in the leptin expression in human adipose tissue *in vitro* (Cullberg et al., 2013) and cultured human adipocytes (Wang et al., 2007). In addition, reduced leptin levels in response to hypoxia were observed not only in human adipocytes (Famulla et al., 2012), but also in animal studies (van den Borst et al., 2013). There are several factors that could affect the results, such as the degree and duration of hypoxia, and subject characteristics.

In human studies, body mass was significantly reduced in lowlanders after hiking or expedition at altitude (Major and Doucet, 2004). Furthermore, fat mass and body weight were reduced to a greater extent in obese persons after several weeks of mild physical exercise in normobaric hypoxia performed at the same relative intensity when compared to normoxia conditions (Wiesner et al., 2010). As indicated in two recent reviews, intermittent hypoxia has been used as an adjunct therapy to enhance weight loss by diet and exercise in obese patients, and an intermittent hypoxia conditioning protocol has been proposed for targeting both weight loss and aerobic capacity improvement (Urdampilleta et al., 2012; Verges et al., 2015). In animal studies, both exercise training and hypoxia significantly reduced body mass, and the additive effects of both treatments were found (Bigard et al., 1991; Chiu et al., 2004). However, both animal studies did not use an obese animal model.

A strength of our current study is to observe the combined effects of continuous hypoxic living (which mimics altitude living in humans) and exercise training on obesity. Another strength is that we directly measured leptin and leptin receptor in intra-abdominal adipose tissue, which can lead to future investigations of underlying mechanisms of the changes in energy regulation.

Although we did see hypoxic living plus exercise training, compared to either hypoxic living alone or living and exercise training in normoxic conditions, resulted in a lower Lee index, we did not see the same group differences in fat pad weights, possibly due to our small sample size. It is a little difficult to interpret the opposite changes in adipose tissue leptin between the SH, TN (slightly decreasing) and TH (significantly increasing) groups; probably, hypoxia, and exercise have a synergistic effect in stimulating leptin production in adipose tissue. Although the trend of changes in serum leptin was similar to that in adipose tissue leptin, no significant group differences were seen in serum leptin; since only one fat pad was collected for measuring adipose tissue leptin, changes in leptin in other fat depots will need to be measured to understand the overall picture and the influence of adipose tissue leptin to the circulating leptin levels. In the current study, food intake and metabolic rate were not recorded, although these factors could potentially affect weight changes in the current study. Additionally, inclusion of a real control group at each time point would further strengthen the study design and help understand our findings.

In conclusion, 3–4 weeks of hypoxic living and treadmill exercise, compared to hypoxic living alone, or living and exercise training in normoxic conditions, resulted in a lower Lee index, and higher adipose tissue leptin/leptin receptor levels in obese rats. Future studies are needed to confirm these findings in human studies and to clarify the mechanisms through which exercise in hypoxic conditions results in changes in body weight and energy-regulating hormones.

## Author contributions

YL, LZ, JX, and ZH acquired the data; YL, LF and TY analyzed and interpreted data; YL and TY drafted the manuscript; YL, MX and LF were responsible for study concept and design; YL and LF obtained research funding.

### Conflict of interest statement

The authors declare that the research was conducted in the absence of any commercial or financial relationships that could be construed as a potential conflict of interest.
